# The Preparation and Characterization of Polylactic Acid Composites with Chitin-Based Intumescent Flame Retardants

**DOI:** 10.3390/polym13203513

**Published:** 2021-10-13

**Authors:** Xiaodong Jin, Suping Cui, Shibing Sun, Jun Sun, Sheng Zhang

**Affiliations:** 1 Faculty of Materials and Manufacturing, Beijing University of Technology, Beijing 100124, China; jinxiaodong@bjut.edu.cn (X.J.); cuisuping@bjut.edu.cn (S.C.); 2 Beijing Key Laboratory of Advanced Functional Polymer Composites, School of Material Science and Engineering, Beijing University of Chemical Technology, Beijing 100029, China; sunj@mail.buct.edu.cn (J.S.); zhangsheng@mail.buct.edu.cn (S.Z.)

**Keywords:** chitin, polylactic acid, flame retardant, mechanism

## Abstract

In this work, a novel intumescent flame retardant (IFR) system was fabricated by the introduction of chitin as a green charring agent, ammonium polyphosphate (APP) as the acid source, and melamine (MEL) as the gas source. The obtained chitin-based IFR was then incorporated into a polylactic acid (PLA) matrix using melt compounding. The fire resistance of PLA/chitin composites was investigated via the limiting oxygen index (LOI), UL-94 vertical burning, and cone calorimeter (CONE) tests. The results demonstrated that the combination of 10%APP, 5%chitin and 5%MEL could result in a 26.0% LOI, a V-0 rating after UL and a 51.2% reduction in the peak heat release rate during the CONE test. Based on the mechanism analysis from both the morphology and the chemical structure of the char, it was suggested that chitin was a promising candidate as a charring agent for chitin reacted with APP and MEL with the formation of an intumescent layer on the surface.

## 1. Introduction

Considering the serious energy crisis, traditional plastics that are synthesized through petroleum-based resources are gradually being replaced by bio-based and biodegradable materials [1]. As an important environmentally friendly polymeric substrate, polylactic acid (PLA), which is obtained from corn, exhibits excellent mechanical properties, transparency, and biological compatibility, but its poor fire resistance restrains its application, especially in the field of overwrapping and electronics and in the electrical industry [2,3,4]. 

Recently, different kinds of flame retardants, including intumescent flame retardants (IFRs), hypophosphites, phytates, metal oxides, nano-particles, and graphite structures, have all been incorporated into PLA matrixes to solve this problem [5,6,7,8,9,10,11]. Among them, IFRs, which consist of an acid source (ammonium polyphosphate (APP)), a charring agent (pentaerythritol (PER)), and a blowing agent (melamine (MEL)), have attracted increasing attention due to their halogen-free and high efficiency characteristics [12]. During combustion, the three components (the acid source, charring agent and blowing agent) react with each other with the formation of an intumescent coating on the surface of a polymeric substrate [13]. As for PLA materials, however, the flame-retardant efficiency of traditional IFR (APP/MEL/PER) is lower than that in other polymer matrixes, which might be due to the deteriorated charring ability of PER [14].

During the past few years, the idea of a bio-based charring agent has come into fashion. Cellulose [15], β-cyclodextrin [16], starch [17], and polypseudorotaxan [18] have all been applied as a charring agent in PLA/IFR composites to improve the fire resistance of PLA and maintain its environmental friendliness at the same time. Among these bio-based charring agents, chitosan (CH), which is extracted from chitin via alkaline deacetylation, has quickly become popular [19]. As a polysaccharide with amino groups and multi-hydroxyl groups, CH has played an important role in IFR systems [20]. Our previous work revealed how modified CH affects the burning behavior of thermoplastic polyurethane (TPU) composites [21]. In addition, CH has been wildly applied in the field of layer-by-layer technology due to its positively charged amino groups. With the combination of some negative charged compounds (such as phytic acid, poly(vinyl sulfonic acid sodium salt) and clay), CH can significantly improve the fire resistance of PU foams, cotton fabrics, and other polymeric substrates with a mere surface coating [22,23,24,25]. In addition to flame retardancy, the incorporation of CH or modified CH with other ingredients also leads to the improvement of various other properties of polymeric substrates (such as UV protection, hydrophilicity and antimicrobial properties) as a number of researchers have recently discovered [26,27,28,29]. 

Though CH has recently enjoyed tremendous popularity as a flame retardant, its matrix, chitin, has not attracted enough attention in the field of fire science. Chitin is also a key polysaccharide and the second most important natural polymer in the world after cellulose, and is mainly extracted from crab and shrimp shells [30]. Once chitin suffers a deacetylation process, CH is obtained. Compared with CH, chitin also exhibits the same multi-hydroxyl groups that are the foundation of chitin’s charring ability. Moreover, chitin also shows a cost advantage because it is directly extracted from natural resources. Therefore, it is of interest to investigate the charring ability of chitin as is done in this work.

In this paper, chitin alone and a novel IFR system consisting of chitin/APP/MEL were introduced into a PLA matrix for the first time. It was expected that chitin could act as a charring agent, which takes effect in the condensed phase through the formation of intumescent char layers, hence improving the fire resistance of the polymeric material. The flame retardancy, thermal stability, and mechanical properties of the PLA/chitin composites were investigated and the flame-retardant mechanism was also proposed.

## 2. Experimental Section

### 2.1. Materials

Polylactic acid (PLA, 3052D) with a specific gravity of 1.24 g/cm^3^ was provided by Natural Works Company. Chitin was obtained from Shandong Luke Chemistry Co., Ltd, Shandong, China. APP (*n* = 1000) was purchased from Shandong Jinyingtai Co., Ltd. MEL was offered by Tianjin Fuchen Co., Ltd, Tianjin, China.

### 2.2. Measurements

Limited oxygen index (LOI) values were measured using a Jiangning JF-3 oxygen index apparatus (Nanjing Jiangning Analytical Instrument Co., Ltd, Nanjing, China) according to ISO 4589. The sample dimensions for the LOI test were 130 mm × 6.5 mm × 3 mm. UL-94 vertical burning experiments were recorded using a Jiangning CZF-3 apparatus (Nanjing Jiangning Analytical Instrument Co., Ltd, Nanjing, China) according to ISO 9773. The sample dimensions for the UL-94 test were 130 mm × 13 mm × 3.2 mm.

The fire performance of the samples was estimated using a cone calorimeter (CONE) (Fire Testing Technology, East Grinstead, UK) according to ISO 5660. The sample dimensions of the CONE test were 100 mm × 100 mm × 3 mm. The heat flux was set at 50 kW/m^2^.

Thermogravimetric analyses (TGA) under nitrogen and air atmospheres were performed using a Q50 apparatus from TA Instruments. The heating rate was 10 °C/min in the range of ambient temperature to 800 °C.

Fourier transform infrared (FTIR) spectra were determined using a Nicolet Nexus 670 FTIR spectrometer. The KBr disk samples were tested with a 1 cm^−1^ resolution over 128 scans, in the range of 4000 to 500 cm^−1^.

X-ray photoelectron spectroscopy (XPS) spectra were recorded using an ESCALAB250 (ThermoVG) spectrometer with Al Kα (1486.6 eV) radiation at a pass energy of 12 kV and 15 mA.

Scanning electron microscopy (SEM) images and energy dispersive X-ray spectroscopy (EDX) tests of char residues of the PLA composites were determined using a Hitachi S-4700 instrument with a voltage of 10 kV. Before tests, all samples were coated with Pt. 

The mechanical properties of the PLA composites were tested via a CMT4104 tensile machine (SANS Company, Shenzhen, China) according to ASTM test methods (D-638) at a speed of 10 mm/min.

### 2.3. Preparation of Flame Retardant PLA Samples

The formulations of the samples are summarized in Table 1. PLA composites were prepared by melt compounding using a micro twin-screw extruder (Wuhan Rayzong Ming Plastics Machinery Co., Ltd, Wuhan, China) at a speed of 50 rpm. The manufacture temperatures were fixed at 160 °C, 180 °C and 175 °C from the hopper to the die, respectively. The resulting extrudates were hot-pressed into standard samples at 180 °C and 10 MPa for 10 min. All materials were dried at 80 °C for 12 h before use.

## 3. Results and Discussion

### 3.1. Flammability 

**Figure 1:**
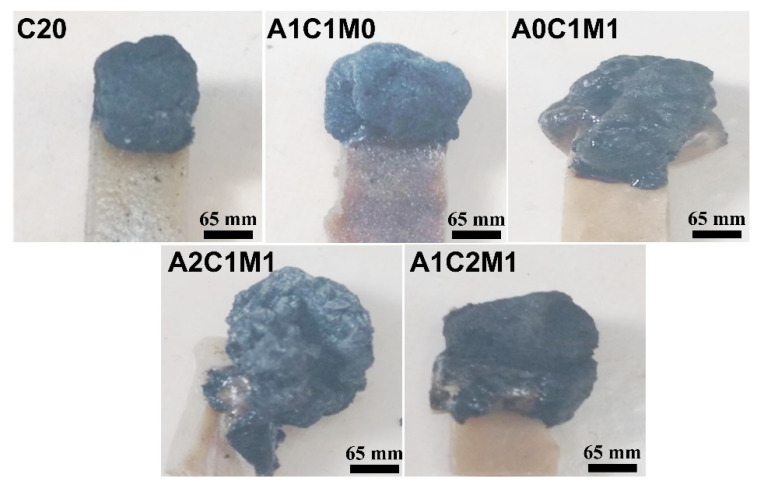
Digital photos of PLA and flame-retardant PLA composites after LOI tests.

The fire testing results, such as the LOI and UL-94 of neat PLA and PLA composites containing chitin, are given in Table 1. In addition, digital photographs of the samples after the LOI tests are shown in Figure 1. For the neat PLA sample, it showed a LOI of 20.0. With the incorporation of chitin alone, the LOI values of the PLA/chitin composites were improved slightly to 24.1 (C15 sample) and 24.5 (C20 sample), respectively. However, chitin is not helpful at upgrading the UL rating for PLA substrate; both the PLA samples with or without chitin shared the same “NR” after the UL-94 test. In spite of this, carbonization of C20 sample after the LOI test was still attractive, as shown in Figure 1. As a result, one can expect that chitin could act well as a charring agent in an IFR system.

Inspired by this idea, chitin combined with an acid source (APP) and a gas source (MEL), was incorporated into a PLA matrix. The fire testing results are also given in Table 1. The mass ratio between these three ingredients is the crucial factor that influences the fire performance of PLA composites. The optical formulation in enhancing the fire resistance of the PLA composites can be obtained when the mass ratio of the APP, chitin, and MEL is set at 2:1:1. At such, not only did the A2C1M1 sample achieve a UL V-0 rating, but it also improved the LOI from 20.0 of neat PLA sample to 26.0. The photos of the PLA samples after LOI may explain the different fire results. One can see from Figure 1 that a well-intumescent char can only be produced for the A2C1M1 sample. 

**Figure 2:**
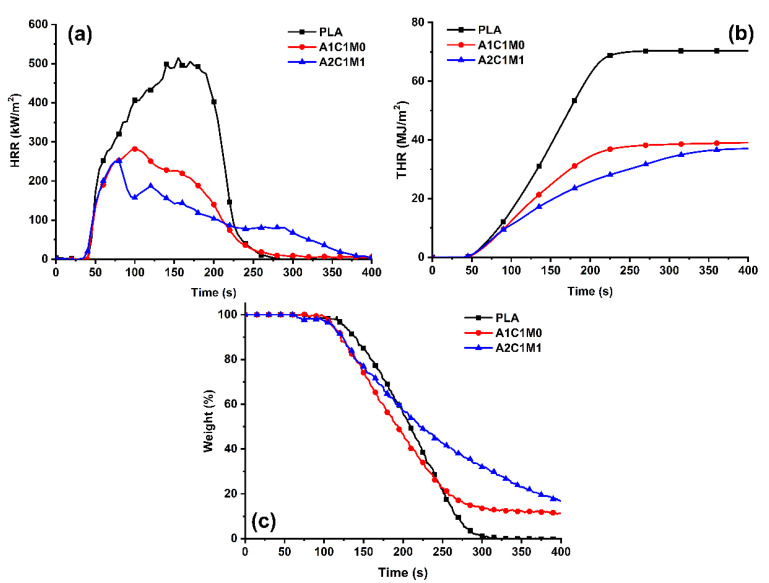
HRR (**a**), THR (**b**) and weight loss (**c**) curves of PLA and PLA composites.

In order to further investigate the flame-retardant effects of chitin-based IFR on PLA composites, CONE tests of neat PLA, A1C1M0, and A2C1M1 samples were conducted, as shown in Figure 2. One can see from Figure 2a that the neat PLA sample exhibited a pHRR value of 514 kW/m^2^. The incorporation of chitin-based IFR greatly decreased the heat release of the PLA materials. By the presence of 20% chitin-IFR, a 45.1% and 51.2% reduction in pHRR could be observed for A1C1M0 and A2C1M1 samples, respectively. A similar tendency could also be found in the THR curves of PLA and PLA composites, as shown in Figure 2b. The THR values were reduced from 71 MJ/m^2^ of neat PLA sample to 39 and 37 MJ/m^2^ for A1C1M0 and A2C1M1 samples, respectively. Moreover, the A2C1M1 sample showed the lowest THR value (49.9% lower than that of neat PLA) among all the materials. It has been reported that the gradient of a THR curve is related to the velocity of flame spread [31]. Compared with the neat PLA sample, the gradient of the THR curves of the flame-retardant PLA composites have obviously been lowered after 75 s. The A2C1M1 sample exhibited the lowest gradient, demonstrating a restriction of flame spread during the fire.

The key data that were collected during the CONE tests are given in Table 2. Unlike other bio-based flame retardants (such as phytic acid [32], chitosan [14] and casein [33]), chitin-based IFR exerts a subtle influence on the TTI value of PLA samples, as the TTI has been shortened slightly from 37 s for neat PLA to 36 and 34 s for A1C1M0 and A2C1M1 samples, respectively. CO and smoke production is closely related to the toxicity of the smoke that evolved during combustion. Therefore, mean COY and TSP values were taken into consideration in this work. For the neat PLA sample, it rarely produces CO and smoke for its mean COY and TSP equal 0.016 kg/kg and 0.16 m^2^, which is consistent with the literature report [34]. However, these values have increased slightly with the incorporation of chitin-based IFR. It is worth noting that the A2C1M1 sample always exhibited a lower mean COY and TSP value compared with those of the A1C1M0 sample.

The weight loss curves of PLA and PLA composites during CONE are shown in Figure 2c. From Figure 2c and Table 2, one can see that neat PLA sample burns out completely without any residues. With the incorporation of chitin-based IFR, the amounts of residues are significantly increased, especially for the A2C1M1 sample (16.16% residues after burning). The proper mass ratio between APP, chitin, and MEL is beneficial for the formation of cohesive, compact, and continuous char residues, which also restrict the production of CO and smoke. 

**Figure 3:**
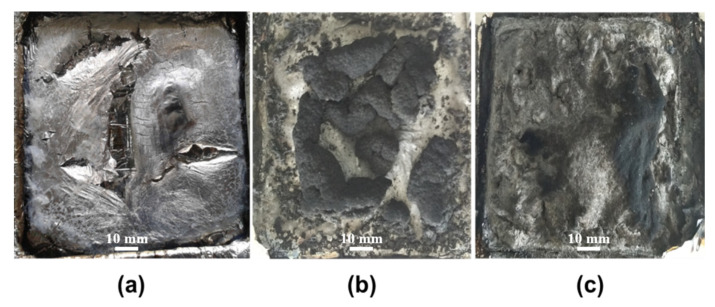
Digital photographs of PLA and PLA composites after CONE test from a vertical perspective: neat PLA (**a**), A1C1M0 (**b**), and A2C1M1 (**c**).

**Figure 4:**
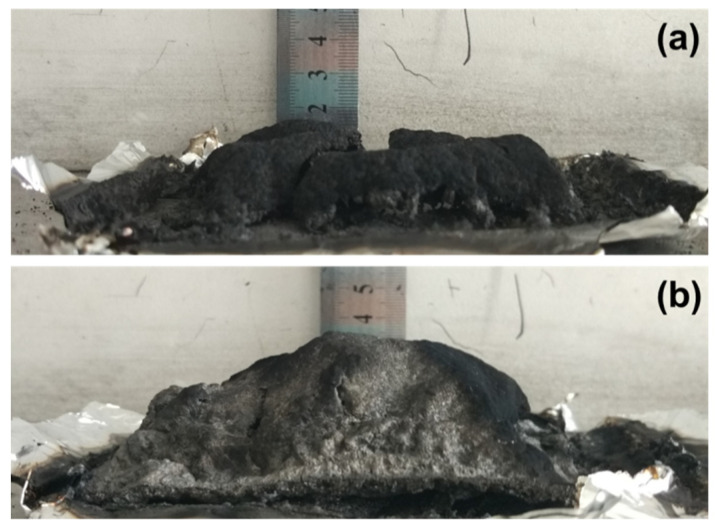
Digital photographs of PLA and PLA composites after CONE test from a horizontal perspective: A1C1M0 (**a**) and A2C1M1 (**b**).

Digital photographs of the residues of the PLA composites containing chitin-based IFR, after CONE tests, are shown in Figure 3 and Figure 4. From the vertical view (Figure 3), one can see that neat PLA sample burns thoroughly. For the A1C1M0 sample, an incomplete char layer with several pieces of swollen residues can be observed. These remains cannot fully cover the surface of the sample, leading to inefficient protection. In the case of the residues produced by the A2C1M1 sample, not only does it strictly enveloped the surface of sample, but it also leads to the highest expansion height after the test (over 3.5 cm), as shown in Figure 4. It has been wildly established that good intumescent behavior of the materials during combustion can only be obtained with matching between the temperature of the gas evolution and the physicochemical properties of the degrading mixtures at the same temperature [35]. In light of this statement, the residues formed by APP and chitin are strong enough to withstand the pressure released by MEL, leading to good intumescent, hence preventing heat transfer and protecting the underlying polymeric substrate.

### 3.2. Thermal Stability 

**Figure 5:**
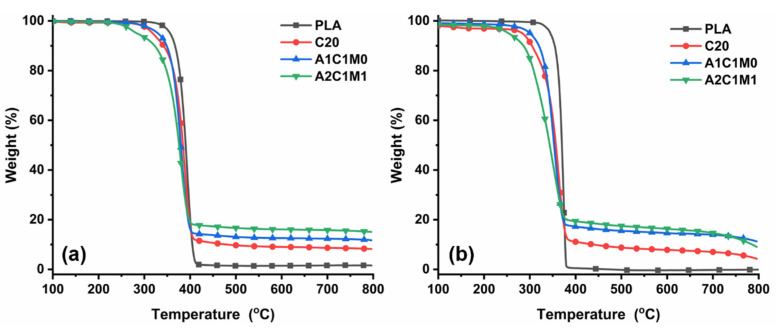
TGA curves of PLA and PLA composites under N_2_ (**a**) and air (**b**) atmospheres.

The TGA curves of PLA and PLA composites under N_2_ and air atmospheres are shown in Figure 5. Under a N_2_ atmosphere (Figure 5a), neat PLA sample exhibited a one-step decomposition routine from 320 to 410 °C. For the C20 and A1C1M0 samples, a two-step degradation process was obtained. The first step around 300 °C corresponded to the separation of the side groups from chitin and/or the releasing of ammonia from APP [36]. The second step resulted in the decomposition of PLA matrix. In the case of the A2C1M1 sample, it also revealed a two-step degradation pathway, but the first step was amplified because of the sublimation of MEL around 300 °C [37]. Compared with the neat PLA sample, the TGA curves of all the PLA composites containing flame retardants were shifted to a lower temperature zone, below 400 °C, suggesting that the incorporation of chitin-based IFRs accelerated the decomposition of polymeric materials. Above 400 °C, on the other hand, PLA composites exhibited a higher thermal stability than neat PLA, demonstrating that the cross-linked networks formed through the interactions of APP, chitin, and MEL further protected the underlying substrate. It can also be seen from Figure 5a that the addition of flame retardants improved the residues of PLA composites at 800 °C through the formation of cross-linking networks by the interaction between polymeric substrates and chitin-based IFRs. The A2C1M1 sample showed the highest char residues of 15.1% among all the materials, clearly suggesting that a proper mass ratio between these three components is beneficial to promoting the charring process of PLA composites. A similar tendency could also be observed for the TGA measurements that are conducted under an air atmosphere, as shown in Figure 5b. The incorporation of chitin-based IFRs accelerated the decomposition of PLA composites below 370 °C, and improved the thermal stability of the polymeric substrate towards higher temperatures due to the formation of a protective layer.

### 3.3. Flame Retardant Mechanism 

**Figure 6:**
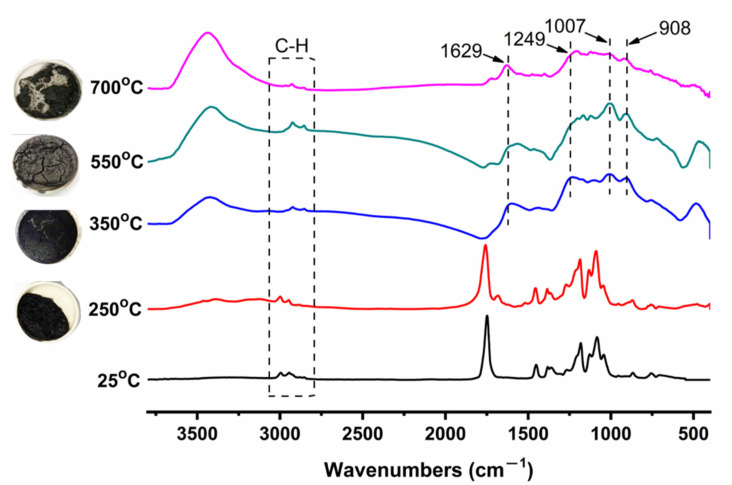
The FTIR spectra of the A2C1M1 sample after calcination in a muffle at different temperatures and their corresponding digital photographs.

The FTIR spectra of the residues of the calcined A2C1M1 sample in a muffle for 30 min, and their corresponding digital photographs, are shown Figure 6. The temperature was set to 250 °C, 350 °C, 550 °C, and 700 °C. At 250 °C, though the sample was in a molten form, the FTIR spectrum of the char remained unchanged compared with that of the A2C1M1 sample at 25 °C. It can be observed that the C–H stretching vibration (2998 and 2947 cm^−1^), the C=O vibration (1757 cm^−1^), the C–H deformation (1456 and 1384 cm^−1^), and the C–O–C vibration (1089 cm^−1^) in Figure 6, conformed with the literature [38]. When the temperature increased to 350 °C and 550 °C, the char exhibited intumescent behavior with some phosphorus containing groups, as revealed in the FTIR spectrum. The peak at 1249 cm^−1^ represents P–O–P cross-linking networks, the peak at 1007 cm^−1^ stands for P–N–C and the peak at 908 cm^−1^ corresponds to P–O. It is well established that P–N–C rich char residues are beneficial for condensed phase protection, resulting in a better flame retardancy [39]. As a result, the characteristic peaks of C–H and C=O from the PLA matrix can still be observed. The residues were gradually degraded when the temperature was further increased to 700 °C. The peak intensity of the phosphorus-containing groups was lowered compared with those at 550 °C as well.

**Figure 7:**
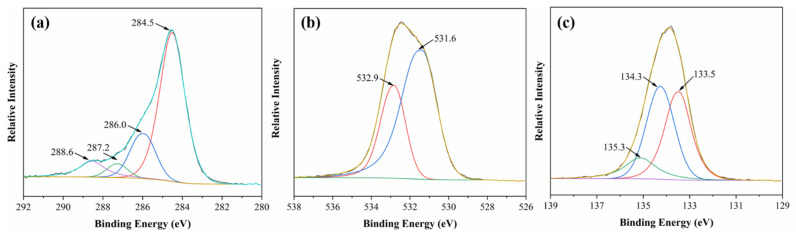
XPS spectra of the residues of the A2C1M1 sample after calcination in a muffle at 550 °C: C1s (**a**), O1s (**b**) and P2p (**c**).

In order to investigate the chemical structure of the char residues, XPS analyses of the residues of the A2C1M1 sample after calcination in a muffle (550 °C for 10 min) were conducted, as shown in Figure 7. For the C1s spectra of the A2C1M1 composites (Figure 7a), the peak at 284.5 eV stands for the C–H and C–C in aliphatic/aromatic compounds, the peak at 286.0 eV is assigned to C–O–C, C–O–P and C–OH, and the peak around 288.6 eV corresponds to the carbonyl groups [40]. Most importantly, the peak which that corresponds to P–N–C can be observed at 287.2 eV. As discussed earlier, the P–N–C–containing structures exhibit positive effects in promoting char formation in the condensed phase [39]. For the O1s spectra (Figure 7b), two peaks can be fitted. The first one at 531.6 eV can be assigned to C=O or P=O, and the second one around 532.9 eV can be assigned to –O– in C–O–C, C–O–P, P–O–P, and/or C–OH groups [41]. The P2p spectra are shown in Figure 7c. The peaks at 134.3 and 135.3 eV correspond to the P–O–C and PO_3_^−^ in phosphate [42]. The other peak at 133.5 eV might be assigned to P–N–C.

**Figure 8:**
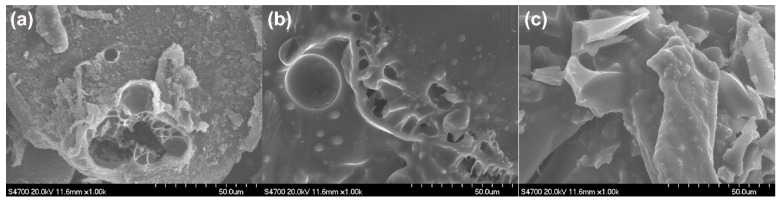
The SEM images of residues of flame-retardant PLA composites collected after the LOI test: C20 (**a**), A1C1M0 (**b**) and A2C1M1 (**c**).

The morphology of the char residues of C20, A1C1M0, and A2C1M1 samples after the LOI test observed using SEM, as shown in Figure 8. The corresponding EDS results of the char are given in Table 3. It can be seen from Figure 8a that the residues formed by the C20 sample exhibited a porous structure that consisted only of C and O, indicating that the acetamido groups were completely dissociated with the release of ammonia. With the combination of APP (A1C1M0, Figure 8b), a much denser residue, which contained 2.89% phosphorus, could be obtained compared with that of C20 sample. By the further incorporation of MEL (A2C1M1), a compact and continuous char could be found, as demonstrated in Figure 8c. It is worth noting that the A2C1M1 and A1C1M0 samples shared the same APP loading (10%). However, the content of phosphorus from the residues obtained after the LOI test of the A2C1M1 sample were greatly increased to 9.8%, indicating that proper concentrations of APP, chitin, and MEL exert positive effects on consolidating the phosphorus in the condensed phase. The increased amounts of phosphide led to highly cross-linked and thermally stable P-containing networks and hence effectively isolated the matrix from heat and oxygen [43].

**Figure 9:**
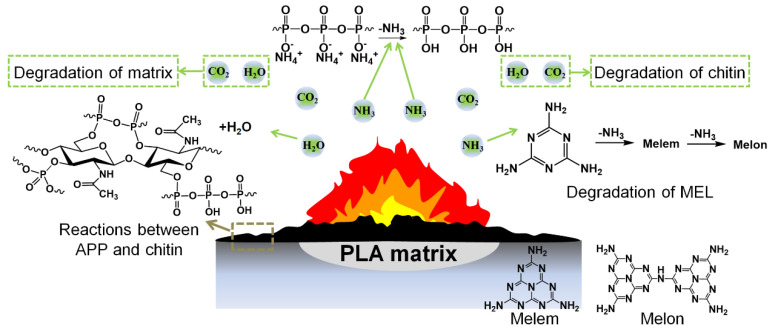
The proposed flame-retardant mechanism of PLA/chitin-based IFR composites.

Based on the discussion above, a flame-retardant mechanism can be proposed, as shown in Figure 9. At first, polyphosphoric acids formed from the degradation of APP with the release of non-flammable gases like ammonia. Meanwhile, ammonia can also be generated by the sublimation of MEL, accompanied by the formation of melem and melon in the condensed phase [44]. All these gases can cause effects in the gas phase through a diluting mechanism. In the condensed phase, polyphosphoric acids react with the hydroxyl groups from chitin through dehydration condensation. The obtained char is further swollen with the release of ammonia from the gas source, leading to the presence of an intumescent protective layer. Then, the degradation of PLA becomes predominant, as does the decomposition of chitin through ring opening in the higher temperature zone, resulting in the formation of a cross-linked structure with aromatic rings.

### 3.4. Mechanical Properties

The mechanical properties of PLA and flame-retardant PLA composites are given in Table 4. For the neat PLA sample, it shows a tensile strength of 59.3 MPa and an elongation at break of 4.1%. After the incorporation of flame retardants, the mechanical properties of the flame-retardant PLA composites decreased dramatically, especially for the A2CAM1 sample, which exhibited the lowest tensile strength (38.9 MPa) and elongation at break (2.1%). Since PLA is very sensitive to acid sources [45], the addition of APP will introduce an unavoidable deterioration in the mechanical performance of PLA composites, as it did in the case of the A2C1M1 sample [46]. However, the tensile strength was still high enough to match requirements in many circumstances.

## 4. Conclusions

In this work, a bio-extractive chitin was used as a novel charring agent, and was combined with APP and MEL. It was suggested that the introduction of chitin-based IFR greatly improved the fire resistance of the PLA matrix. With the combination of 10%APP, 5%chitin and 5%MEL, the LOI value of the PLA composites increased to 26.0%, the UL rating was upgraded to V-0 rating, the pHRR value was decreased to 251 kW/m^2^, and the residue amount also increased from 0.0% to 14.8% at 700 °C under an air atmosphere. Through the morphology and the chemical structure of the char, the charring ability of chitin and its reactivity with APP/MEL has been clearly demonstrated. 

## Figures and Tables

**Table 1:** Formulations and flammability of PLA and flame-retardant PLA composites.

Samples	PLA (wt%)	Chitin (wt%)	APP (wt%)	MEL (wt%)	LOI (%)	UL-94
PLA	100	0	0	0	20.0	NR ^b^
C15	85	15	0	0	24.1	NR
C20	80	20	0	0	24.5	NR
A1C1M0 ^a^	80	10	10	0	27.2	V-2
A0C1M1	80	0	10	10	19.6	V-2
A2C1M1	80	5	10	5	26.0	V-0
A1C2M1	80	10	5	5	22.3	V-2

^a^ A = APP, C = chitin, M = MEL; A1C1M0 means A:C:M = 1:1:0 (mass ratio). **^b^** NR = no rating.

**Table 2:** CONE test results of PLA and PLA composites.

Samples	pHRR (kW/m^2^)	TTI (s)	THR (MJ/m^2^)	Mean COY (kg/kg)	TSP (m^2^)	Char Residues (%)
PLA	514 ± 36	37	71 ± 5	0.016	0.16	0.0
A1C1M0	282 ± 20	36	39 ± 3	0.026	1.75	11.3
A2C1M1	251 ± 17	34	37 ± 3	0.021	0.48	16.6

pHRR = peak heat release rate, TTI = time to ignition, THR = total heat release, Mean COY = mean CO yield, TSP = total smoke production.

**Table 3:** EDX results of residues for flame-retardant PLA composites after LOI test.

Samples	C (wt%)	O (wt%)	N (wt%)	P (wt%)
C20	81.35	18.65	-	-
A1C1M0	49.90	47.21	-	2.89
A2C1M1	39.16	36.60	14.44	9.80

**Table 4:** Mechanical properties of PLA and flame-retardant PLA composites.

Samples	Tensile Strength (MPa)	Elongation at Break (%)
PLA	59.3 ± 3.2	4.1 ± 0.3
C20	42.7 ± 2.9	2.5 ± 0.2
A1C1M0	40.1 ± 2.1	2.4 ± 0.1
A2C1M1	38.9 ± 2.5	2.1 ± 0.2

## Data Availability

Not applicable.
